# Bilateral Kohler's Disease: A Case Report

**DOI:** 10.7759/cureus.44929

**Published:** 2023-09-08

**Authors:** Sanjay V Deshpande, Radha A Channawar, Hitendra Wamborikar, Bhushan Patil, Aditya Pundkar

**Affiliations:** 1 Orthopedics, Jawaharlal Nehru Medical College, Datta Meghe Institute of Medical Sciences, Wardha, IND

**Keywords:** navicular, avascular necrosis, pediatric population, pain, kohler's disease

## Abstract

Kohler’s disease is characterized by osteochondritis of the navicular bone due to various factors like the lack of blood supply and late ossification of the bone. In particular, it is a disease of the pediatric age group, which has male preponderance. It may present with bony pain unilaterally or, at times, be asymptomatic and diagnosed accidentally. Clinical presentation and radiological investigations are the mainstay of diagnosis. This self-limiting condition requires only symptomatic conservative management. A surgical approach is not yet indicated.

One such case of bilateral Kohler’s disease is presented in this report. Here, we discuss the disease's presentation, examination, treatment, and prognosis.

## Introduction

Kohler’s disease is characterized by avascular necrosis of the navicular bone of the foot. It may arise due to compression of the bone or due to any physical trauma to the site [1,2]. It is a disorder predominantly seen in the pediatric male population. Around two percent of the pediatric population has been estimated to present with Kohler's disease [1,3]. The patient presents with pain, swelling over the area of the navicular bone, and limping gait due to pain along the arch [1-4]. This disease usually presents unilaterally and is observed to be self-limiting in most of the cases [5]. Many etiological factors can lead to the osteochondrosis of the navicular bone, which is the last bone to ossify in the foot and a crucial part of weight bearing and the vascular supply of the bone [1,2,6,7]. The bony changes like sclerosis, irregular border, and hyper-dense bone can be visualized on radiographic imaging [4,6,8]. The disease management includes symptomatic treatment and a short leg cast as per requirement [1,3,6,9].

## Case presentation

The CARE checklist has been followed in reporting this case.

History

The patient’s history was narrated by his father. A six-year-old male child presented with a painful right foot for two days. The child had a history of twisting the right ankle and foot (inversion and plantar flexion of ankle and foot) while playing. The child came home limping on the right foot. Pain developed immediately after the twist, along with swelling. The child was unable to bear weight on his right foot. Pain was sharp shooting and was aggravated by movements and relieved by rest. Weight-bearing was painful. The child could not carry out his day-to-day activities and even needed to be carried for toilet activities.

Examination

The six-year-old child came to the outpatient department in a wheelchair. Gait is antalgic. Weight-bearing on the right foot is painful, and the patient seeks some support to shed the load on his right foot, and ankle. When asked to walk, he walked by shifting the weight on the lateral side of his foot. The right foot and ankle were held in hundred degrees of plantar flexion with the forefoot adducted and inverted. There was diffuse swelling on the anterior aspect of the ankle and dorsum of the foot. The local temperature was normal. Tenderness over the anterior aspect of the ankle joint extends up to the base of the first metatarsal on the dorsal side.

Movements 

Ankle dorsiflexion was found to be painful with restriction of dorsiflexion after ninety degrees. Plantar flexion has painful restriction after hundred degrees. Dorsalis pedis pulsations were palpated and equal on both sides. Extensor hallucis longus and extensor digitorum power is normal. On examination of the left foot, it was normal and asymptomatic.

X-ray

**Figure 1:**
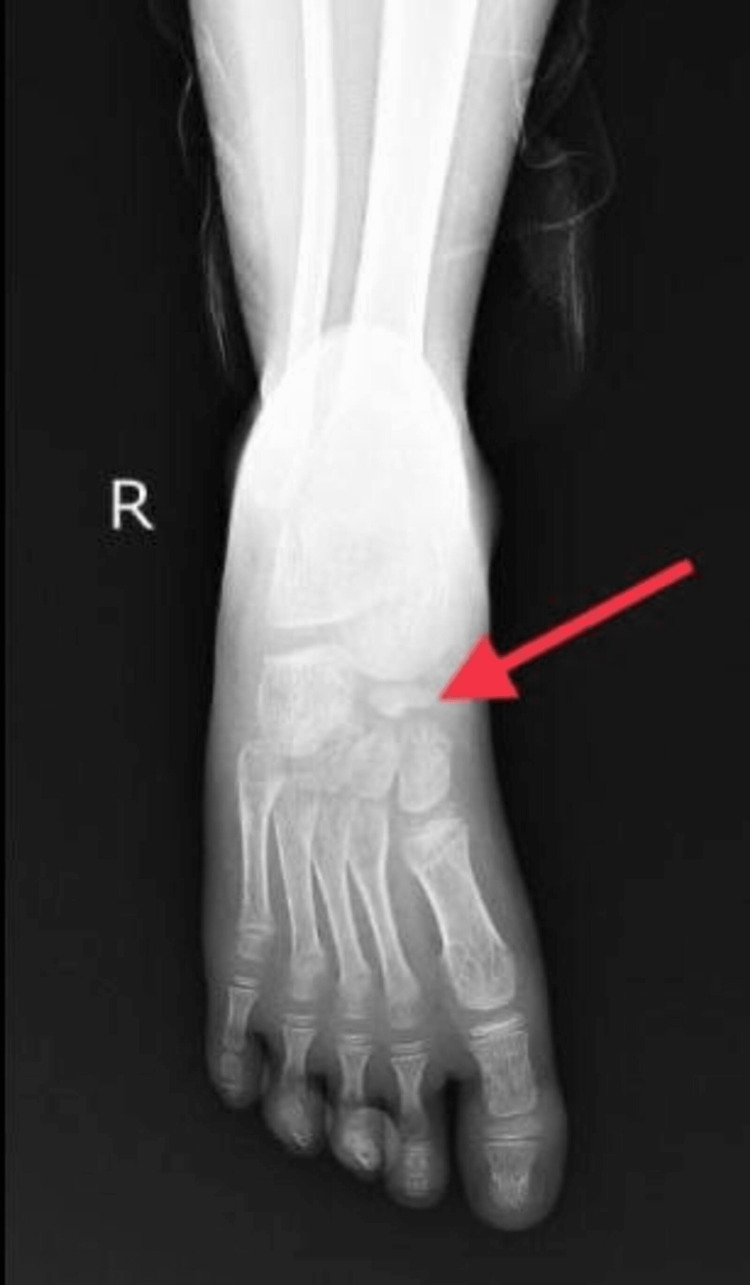
X-ray anteroposterior view of the right foot and ankle

**Figure 2:**
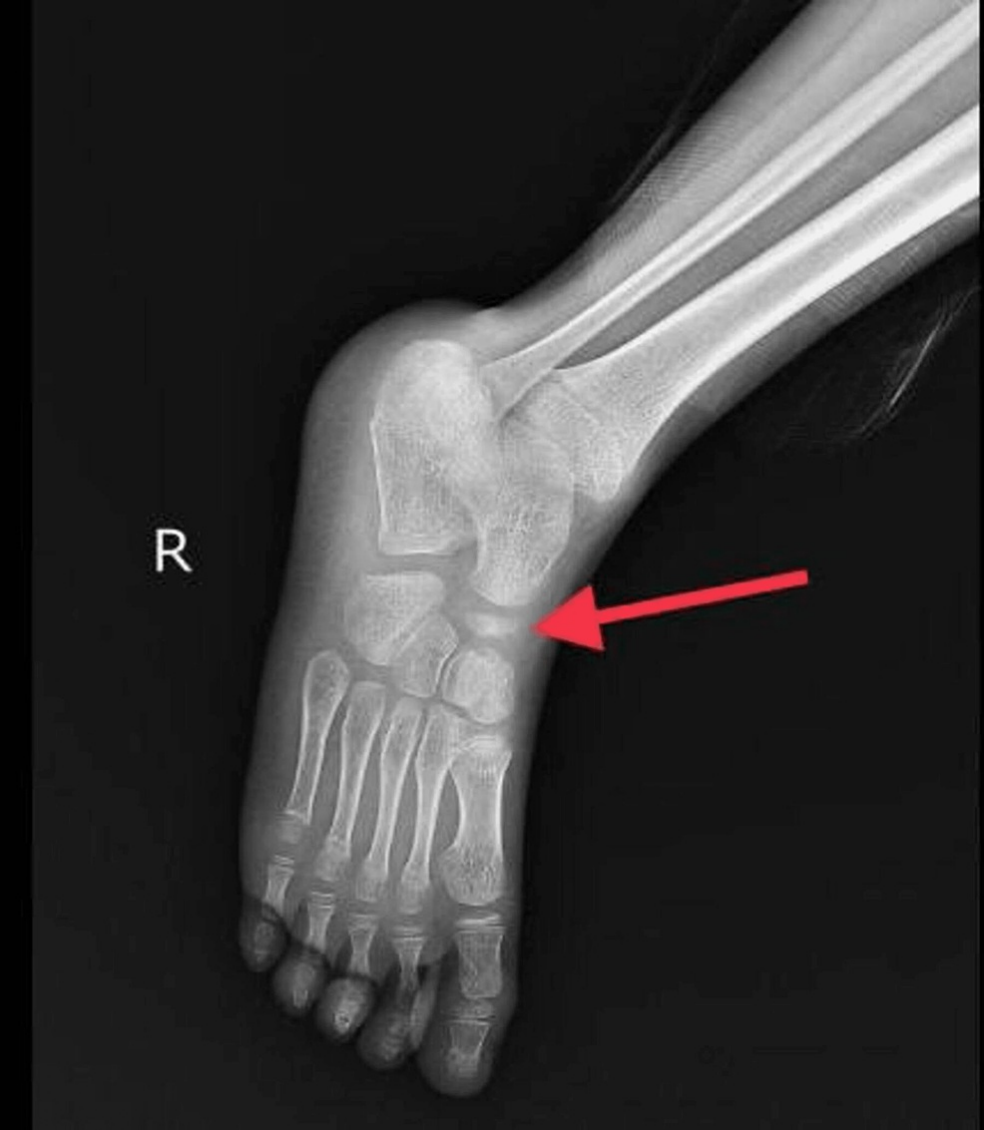
X-ray oblique view of the right foot and ankle

The following findings were seen: On the anteroposterior and oblique view of the right foot, irregular, sclerosed, and increased density of the navicular bone is observed, as seen in Figures 1, 2.

**Figure 3:**
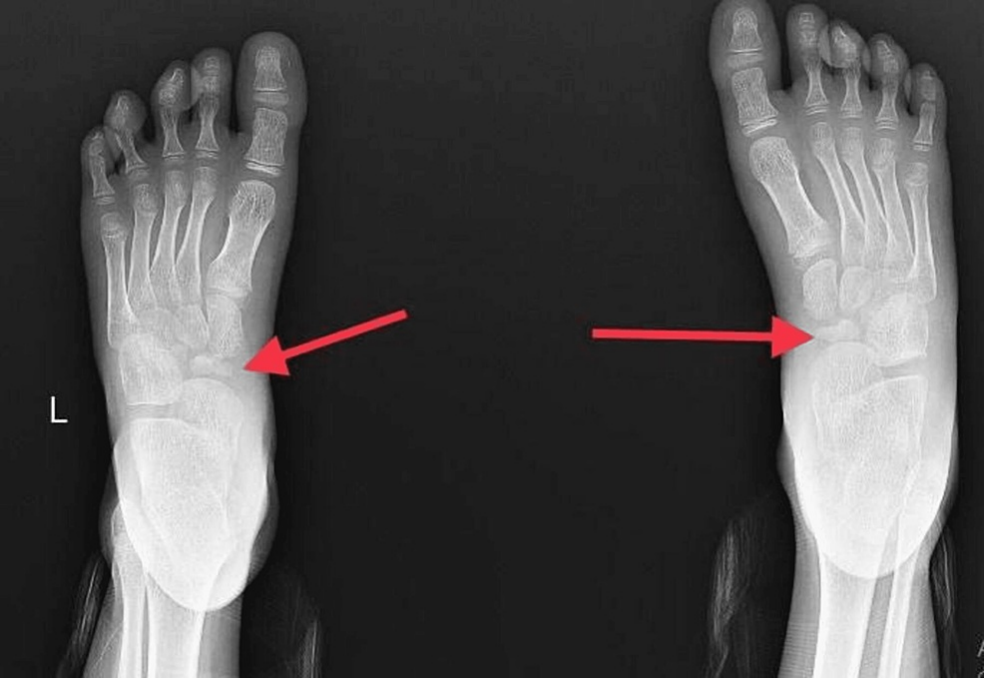
X-ray anteroposterior view of the left and right feet and ankle

On the anteroposterior view of the left and right feet, the navicular bone in both feet was irregular, shrunken, and hyper-dense, as seen in Figure 3.

Talus, calcaneum, cuneiforms and all other tarsals, and metatarsals were normal.

Diagnosis

According to the patient’s history, examination, and radiological findings, this is a case of Bilateral Kohler’s disease.

Treatment

The patient was given a below-knee plaster splint for three weeks.

## Discussion

Etiopathogenesis

**Figure 4:**
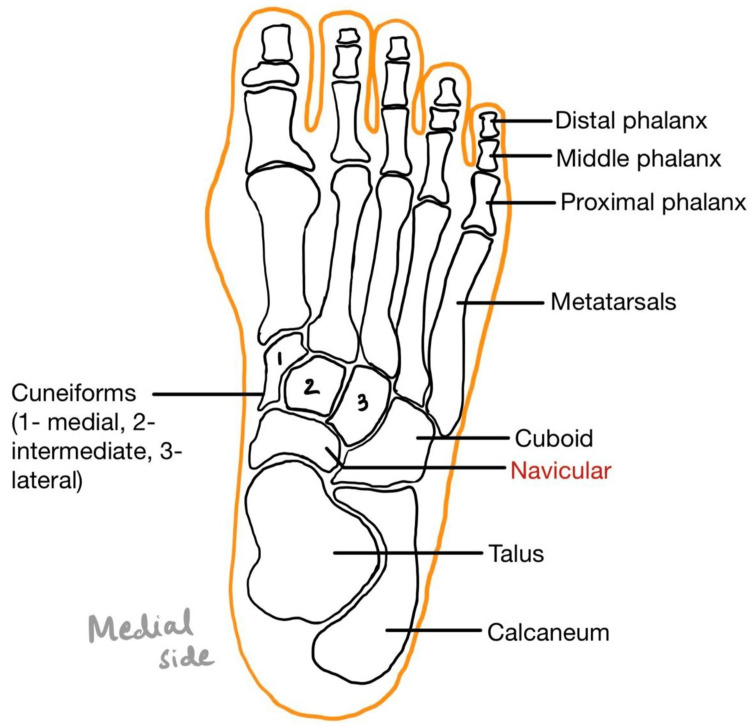
Bony anatomy of the foot The diagram is made by the author

**Figure 5:**
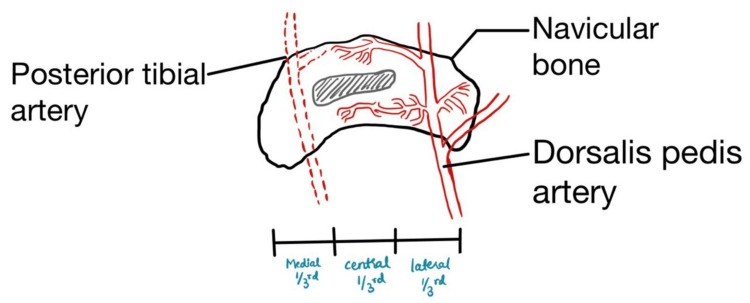
Blood supply and regions of navicular accordingly The diagram is made by the author

The articulation of various bones and cartilage forms the foot. One of them is the navicular bone (Figure 4). This small boat or pyriform-shaped bone plays a crucial role in body weight bearing, adduction and abduction of the midfoot, and maintaining the medial arch of the foot [2,8]. It receives blood supply from two vessels, based on which it can be divided into three regions - medial, central, and lateral (Figure 5). The lateral aspect receives supply from the dorsalis pedis artery, while the medial aspect receives supply from the artery arising from the medial plantar arch from the posterior tibial artery [1,2,10]. This leads to the formation of an avascular or blood-compromised zone in the center. Although a few vascular foramina supply the central zone, they are susceptible to compression [1,2,6]. Another quoted etiology for Kohler’s disease is the ossification process of the bone. The navicular bone is the last bone to ossify in the foot, with multiple ossification centers that further make the bone susceptible to the effects of weight bearing and trauma [1,2]. Navicular bone ossifies enchondrally at a later age in boys than girls; thus, with increasing weight and activities, it is bound to be compressed between the already ossified talus and calcaneum bones [3,4,8].

Clinical features

In the case of osteochondrosis, the case may present with gradual pain in the ankle over the area of navicular bone - dorsomedial aspect of the foot, swelling, erythema, and limping gait. Limping or antalgic gait is characterized by walking with weight bearing on the lateral side of the foot [1,4-6,8-10]. History of trauma may or may not be present, or there can be an exacerbation after injury [4,6-8]. Sometimes, the patient could be asymptomatic, and the diagnosis is made accidentally [1,2,5]. Most cases have presented with unilateral Kohler's disease; bilateral findings are a rarer possibility [1,2,4,8].

Examination findings

Tenderness, swelling, rubor, and calor can be present over the dorsomedial aspect of the foot pertaining to osteochondritis. Pain on adduction and abduction of midfoot [1,2,4,7,8].

Investigations

There are no significant findings in inflammatory blood markers. However, they can be used to rule out any infectious cause (leukocyte counts, packed cell volume, erythrocyte sedimentation rates, or C-reactive proteins) [1,7].

Radiologic investigations show thinned-out navicular bone, fragmentation with loss of trabecular pattern of bone, and sclerosed and hyper-dense bone. Surrounding soft tissue swelling can also be seen [1-4,6,9,11]. The shape of the bone might be distorted or conserved in cases of uniform sclerosis. Variation in sclerosis directs to loss of vasculature from a single vessel or both. Uniform sclerosis points toward loss of blood supply from a single vessel, whereas patchy sclerosis points towards loss of blood supply from multiple vessels [2,4,8]. There can also be flattening of navicular bone seen [3,4,9]. Bone scans, scintigraphy, computed tomography, and magnetic resonance imaging (MRI) are not much beneficial in planning the trajectory of the disease. However, MRI can depict tarsal involvement and edematous navicular bone [7,8,10].

Differential diagnosis

Due to its presenting complaints, Kohler's disease can be compared with many conditions that present with painful movements, and changes in gait. Some of these include infection, stress fractures, tarsal coalition, tendonitis, and inflammatory arthritis. However, they can be ruled out by age of presentation, blood, and radiographic investigations [1,2,7]. 

Treatment

Measures to treat Kohler’s disease include a non-surgical approach in a wide range of cases. Usually, the condition is found to be self-limiting but symptomatic treatment for pain such as non-steroidal anti-inflammatory drugs and rest to avoid weight bearing can be advised [1-3,6,11]. However, there are other modalities like applying a short leg cast for around six weeks, using rigid sole shoes, giving arch supports, and non-weight bearing crutches [1-3,6,7,10,11].

Prognosis and complications

Attributing to proper management, follow-up, and radial blood supply of navicular, Kohler’s disease has a positive prognosis. Normal anatomy of the bone can be restored [1-3,6,7]. Complication like the talonavicular coalition is an infrequent possibility [2,10].

## Conclusions

In a nutshell, Kohler's disease is an osteochondritic compromise of the bone arising from the interplay of many factors. Weight-bearing gets laborious as the bone weakens. The movement of ankle gets restricted due to collapse of navicular bone. The child has an antalgic limping gait. The presentation may be preceded by trauma or can be an accidental diagnosis. Infection and other differentials like fracture must be ruled out. Management is symptomatic that includes pain relief and short leg casting. Overall prognosis is good owing to proper rest for the blood supply of navicular to re-establish. in this article, we have presented a case of bilateral Kohler's disease which is not found occasionally.
